# COVID-19: Unmasking Emerging SARS-CoV-2 Variants, Vaccines and Therapeutic Strategies

**DOI:** 10.3390/biom11070993

**Published:** 2021-07-06

**Authors:** Renuka Raman, Krishna J. Patel, Kishu Ranjan

**Affiliations:** 1 Department of Surgery, Weill Cornell Medical College, New York, NY 10065, USA; rer2029@med.cornell.edu; 2 Mount Sinai Innovation Partners, Icahn School of Medicine at Mount Sinai, New York, NY 10029, USA; krishna.patel2@mssm.edu; 3 School of Medicine, Yale University, New Haven, CT 06519, USA

**Keywords:** SARS-CoV-2, COVID-19, variants, vaccines, immune dysregulated, comorbidities, antibody, spike protein, variants of concern (VOC), biomolecules, coronavirus

## Abstract

Severe acute respiratory syndrome coronavirus 2 (SARS-CoV-2) is the etiological agent of the coronavirus disease 2019 (COVID-19) pandemic, which has been a topic of major concern for global human health. The challenge to restrain the COVID-19 pandemic is further compounded by the emergence of several SARS-CoV-2 variants viz. B.1.1.7 (Alpha), B.1.351 (Beta), P1 (Gamma) and B.1.617.2 (Delta), which show increased transmissibility and resistance towards vaccines and therapies. Importantly, there is convincing evidence of increased susceptibility to SARS-CoV-2 infection among individuals with dysregulated immune response and comorbidities. Herein, we provide a comprehensive perspective regarding vulnerability of SARS-CoV-2 infection in patients with underlying medical comorbidities. We discuss ongoing vaccine (mRNA, protein-based, viral vector-based, etc.) and therapeutic (monoclonal antibodies, small molecules, plasma therapy, etc.) modalities designed to curb the COVID-19 pandemic. We also discuss in detail, the challenges posed by different SARS-CoV-2 variants of concern (VOC) identified across the globe and their effects on therapeutic and prophylactic interventions.

## 1. Introduction

**Figure 1:**
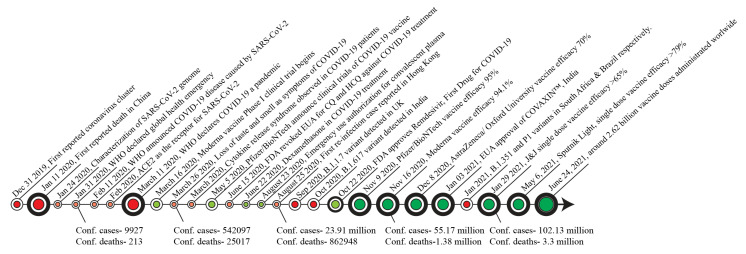
Timeline of major key events in the progression of the COVID-19 pandemic and vaccine development. Counts shown here are confirmed cases and deaths worldwide (https://ourworldindata.org/- Source- Johns Hopkins University CEES COVID-19 DATA, accessed date: 28 May 2021). CQ, Chloroquine; HCQ, Hydroxychloroquine; EUA, emergency use authorization.

The catastrophic spread of coronavirus disease 2019 (COVID-19) has already claimed millions of lives across the globe and has been declared a public health emergency of international concern by the World Health Organization (WHO) [1] (Figure 1). So far, there are seven different types of coronaviruses documented. Among these, four common human coronaviruses—229E, NL63, OC43 and HKU1—cause mild infections [2]. However, individuals infected with either of the other three coronaviruses—severe acute respiratory syndrome coronavirus (SARS-CoV), Middle East respiratory syndrome coronavirus (MERS-CoV) and SARS-CoV-2—develop severe respiratory distress and viral pneumonia and may ultimately succumb to the disease [3,4,5]. SARS-CoV-2, the causative agent of the ongoing COVID-19 pandemic, is a newly identified, highly diverse, enveloped single-stranded RNA virus [4,5,6,7]. It is noteworthy that the nucleotide sequence of SARS-CoV-2 nearly matches (96% similarity) with a bat coronavirus RaTG13 (GenBank: MN996532.1), suggesting the possibility of bats as the most likely progenitors of SARS-CoV-2 and the source for zoonotic spillover to human [5,8]. 

**Figure 2:**
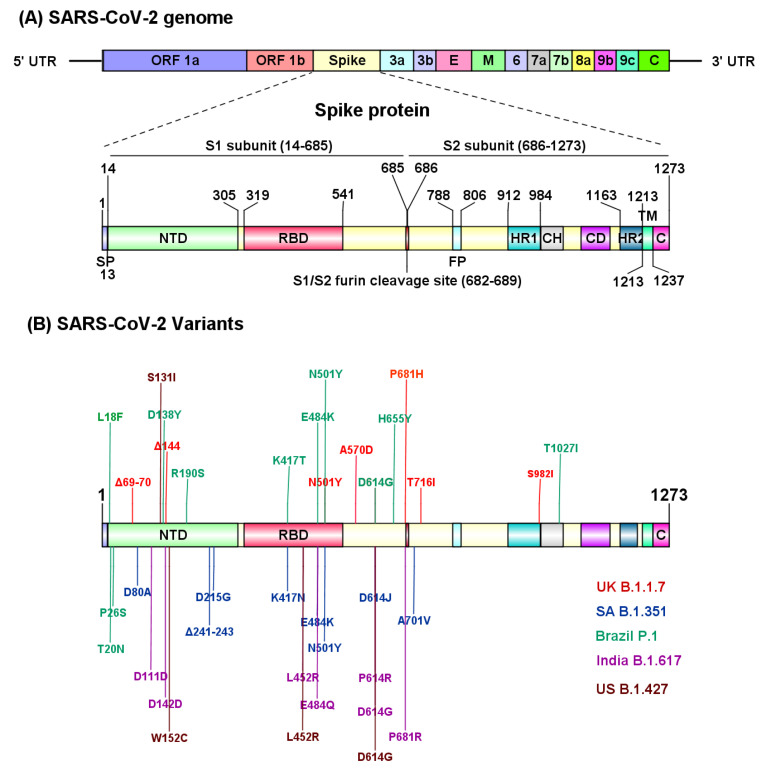
Structural regions of SARS-CoV-2 involved in the pathogenicity. (**A**) SARS-CoV-2 genomic organization and structural components of spike (S) protein. The furin cleavage site (682–689 residues) at the junction of S1 and S2 subunit is critical to facilitate viral fusion and entry to host cells. (**B**) SARS-CoV-2 variants with identified mutation sites in the structural region. UTR, untranslated region; SP, signal peptide; FP, fusion peptide; HR, heptad repeat domain; TM, transmembrane domain; CP, cytoplasmic domain. UK, United Kingdom; SA, South Africa; US, United states. This figure was prepared using IBS 1.0.3 [31].

The molecular characterization through an RNA-based metagenomic next-generation sequencing (mNGS) analysis revealed that the SARS-CoV-2 genome is 29,881 bp in length (GenBank no. MN908947) and encodes 9860 amino acids [9]. The SARS-CoV-2 genome encodes distinct structural and nonstructural proteins. Genes encoding, spike (S) glycoprotein, envelope (E) glycoprotein, membrane (M) glycoprotein and nucleocapsid (N) protein constitute the structural components, whereas 3-chymotrypsin-like protease, papain-like protease, and RNA-dependent RNA polymerase, in addition to several accessory proteins, constitute the nonstructural framework of SARS-CoV-2 [10] (Figure 2A). The S glycoprotein is composed of 1273 amino acids, including the N terminal signal peptide (SP, 1–13 residues), the S1 (14–685 residues) and S2 (686–1273 residues) subunits. Furthermore, the S1 subunit contains an N-terminal domain (NTD, 14–305 residues) and a receptor binding domain (RBD, 319–541 residues), while the S2 subunit is composed of the fusion peptide (FP, 788–806 residues), heptapeptide repeat sequence 1 (HR1) (912–984 residues), HR2 (1163–1213 residues), TM domain (1213–1237 residues) and cytoplasm domain (1237–1273 residues) [11] (Figure 2A). The S1 and S2 subunits are critical in assembly and surface projection of the S protein, which interacts with cognate Angiotensin-Converting Enzyme 2 (ACE2) receptors expressed on the lower respiratory pneumocytes of the host [5,12]. The S protein is cleaved by host transmembrane Serine Protease 2 (TMPRSS2), into the S1 subunit and S2 subunit at the furin cleavage site (682–689 residues), to facilitate viral fusion and entry [13,14] (Figure 2A). Post intracellular entry, SARS-CoV-2 hijacks the host cell machinery to rapidly synthesize viral envelope, nucleocapsid, and the replicase polyproteins to assemble and release virus progenies [15,16]. Recent studies have identified several SARS-CoV-2 variants (B.1.1.7, B.1.351, P.1, B.1.617, CAL.20C) carrying deleterious mutations in the S protein that evade host immune recognition, which further exacerbate the pathogenicity and transmission of COVID-19 [4,5,8] (Figure 2B). Molecular characterization of different SARS-CoV-2 variants is imperative to determine the transmission rate and further identify target sites to develop effective therapies for COVID-19.

The infection and pathogenicity of SARS-CoV-2 in humans was initially reported in the lung [3], but further studies identified SARS-CoV-2 infection vulnerability to other organs, including liver, brain, kidneys and intestine [13,17,18,19,20]. Studies reported that an average incubation period of SARS-CoV-2 in the host is approximately 4–5 days [6,7,21,22] followed by onset of symptoms in 11–12 days [23]. Notably, in some cases, the SARS-CoV-2-infected patients may remain completely asymptomatic but could potentially transmit the virus [24,25]. The severely infected COVID-19 patients develop acute respiratory distress syndrome (ARDS, a common clinical complication associated with viral pneumonia and hypoxemia [26,27,28,29,30]. Given the fact that severe COVID-19 illness corresponds to altered immune response and exaggerated cytokine storm, it is important to understand and design a better treatment approach for patients with pre-existing immunological comorbidities, such as autoimmune diseases and cancer.

Finally, the COVID-19 pandemic has led to the approval of novel vaccine candidates at an unprecedented pace. The pandemic has seen the emergence of nucleic acid vaccines as promising alternatives to conventional vaccine approaches, while the development of effective antiviral therapies for treating SARS-CoV-2 infection are in progress. In this review, we discuss the effect of SARS-CoV-2 infection in patients with altered immune responses, emergence of novel SARS_CoV2 variants, as well as leading therapeutic approaches and vaccines in development to curb the COVID-19 pandemic.

## 2. The Pathophysiology of COVID-19 in Immune-Dysregulated Patients

The severity of SARS-CoV-2 infection appears to be exacerbated by both viral infection and a hyperactive immune response in the host. Based on available cohort, case-control, or cross-sectional studies, it is now evident that patients with underlying medical conditions are more susceptible to COVID-19 related morbidities and mortalities. Importantly, in-depth studies are needed to understand COVID-19 disease etiologies and common therapies used to treat patients with pre-existing comorbidities. In critically ill COVID-19 patients, a hyperactivation of proinflammatory cytokine (cytokine storm) phase with subsequent tissue damage, contributes to the exacerbation of comorbidities [32]. An aberrant elevation of different inflammatory mediators, including IL-1β, IL-1RA, IL-8, IL-9, IL-7, IL-10, fibroblast growth factor (FGF), granulocyte-macrophage colony-stimulating factor (GM-CSF), granulocyte-colony-stimulating factor (G-CSF), IFNγ, interferon-γ-inducible protein (IP10), monocyte chemoattractant protein (MCP1), platelet-derived growth factor (PDGF), macrophage, inflammatory protein 1 alpha (MIP1A), vascular endothelial growth factor (VEGF) and tumor necrosis factor (TNFα), contribute to the severity of COVID-19 [27,33]. Most importantly, terminally ill patients with elevated IL-6 levels succumb to COVID-19 more readily than survivors [34]. Therefore, an awry immune response may be a critical risk factor in individuals with pre-existing morbidities. Furthermore, there is limited data available to understand the impact of emerging SARS-CoV-2 variant related pathogenicity on the progression of mentioned comorbidities. Some of the chronic illnesses associated with inflammatory dysregulation will be further analyzed in this section.

### 2.1. Multiple Sclerosis (MS)

MS is the most predominant chronic inflammatory disease of the central nervous system (CNS), affecting the brain and spinal cord [35]. In a large cohort study, it was found that the prevalence of COVID-19 associated death was proportionally correlated with chronic neurological manifestation, including MS [36]. Furthermore, a small cohort study reported that there was a 2.5 times higher incidence measure of COVID-19 in patients with MS compared to the general population [37]. A recent global cohort study (representing 13 countries and 4 continents) reported neurological symptoms, including headache and anosmia, in COVID-19 patients, and those patients with clinical neurological symptoms were more susceptible to in-hospital death [38]. In contrast, two independent studies reported that patients with MS are less likely to be susceptible to COVID-19 infection [39,40]. Therefore, more comprehensive, and mechanistic studies are required to further evaluate the aggression of COVID-19 related immune dysregulation in MS patients, that will help in designing therapeutic strategies.

### 2.2. Rheumatoid Arthritis (RA)

RA is a chronic inflammatory disease of the joints involving inflammation of the synovial membrane, leading to damage of articular cartilage and juxta-articular bone [41]. Patients with RA are more susceptible to respiratory illness, osteoporosis, infection and cancer [42]. A retrospective case-control study conducted in Korea at the national level found high susceptibility of SARS-CoV-2 infection in RA patients [43]. An interesting study suggested that the array of proinflammatory cytokines elevated in COVID-19 patients are potential targets in the treatment of RA [44]. In contrast, Monti S. et al. conducted a retrospective study on 320 patients treated with antirheumatic drugs. They found no risk of respiratory complications from SARS-CoV-2 in 4 clinically confirmed COVID-19 patients compared to the general population [45]. Given the relevance of immune disorder in the pathogenesis of RA, further studies warrant reporting the clinical manifestation of RA in COVID-19 patients, which will help in management and treatment of this disease.

### 2.3. Systemic Lupus Erythematosus (SLE)

SLE is a chronic multisystem autoimmune disorder characterized by generation of antibodies to self-antigens, leading to altered immune tolerance, tissue, and organ damage [46]. Notably, viruses tend to mitigate antiviral interferon (IFN) response to escape innate immune recognition [47], and an increased level of type I IFN in SLE patients may provide some degree of protection against SARS-CoV-2 infection [48]. Some earlier studies aimed to investigate if SARS-CoV-2 infection exacerbated SLE disease prevalence, however they have not found a substantial vulnerability of COVID-19 in infected SLE patients [49,50]. A small cohort study by Fernandez-Ruiz et al. reported that COVID-19 confirmed SLE patients have a higher hospitalization rate compared to the general population, independent of mortality rate [51]. The immunosuppressive treatment in SLE patients has not shown a higher rate of SARS-CoV-2 infection or COVID-19 symptoms [52]. Still further studies with large cohort size are needed to understand the impact of SARS-CoV-2 infection on SLE patients, which will further help in designing strategies to contain COVID-19 in SLE patients.

### 2.4. Cancer

The immunological manifestation of cancer is frequently associated with infiltration of immunosuppressive leukocytes, hyperactivation of immunosuppressive cytokines (e.g., TGFβ), suppressive proinflammatory signals and impaired dendritic cell maturation [53]. Studies reported that cancer patients are more susceptible to risk of contracting the virus and developing COVID-19 [54,55]. Furthermore, a retrospective study analyzing data sets from the COVID-19 and Cancer Consortium (CCC19) registry showed that the all-cause mortality among cancer patients was highly associated with COVID-19 progression [56]. Importantly, cancer patients undergoing chemotherapy and surgery develop an immunocompromised condition and are at a higher risk of contracting COVID-19 infection [57,58]. Immune checkpoint inhibitors (ICI) and CAR-T cell mediated cancer immunotherapy usually lead to hyper activation of IL-6, IFN- γ and other cytokines, causing severe illness and death [59], a condition similar to that observed in severe COVID-19 patients [59]. In this context, an elevated level of proinflammatory CCR6^+^ Th17 in CD4^+^T cells and hyperactivated cytotoxic CD8+T cells was observed in confirmed COVID-19 patients, suggesting that pathologic hyperactivation of the immune response contributes to severe immune injury [60]. Therefore, a strong disease management and precautionary treatment protocol needs to be defined to protect cancer patients from COVID-19 associated inflammatory injury.

### 2.5. Inflammatory Bowel Disease (IBD)

Critically ill patients with COVID-19 show gastrointestinal (GI) complications including diarrhea, nausea, vomiting and abdominal pain during their stay at the hospital [61,62], thereby demonstrating that GI complications may aggravate disease severity. Furthermore, Lin et al. reported the presence of SARS-CoV-2 RNA in the specimens of oesophagus, stomach, duodenum and rectum from patients with severe COVID-19 disease [63], suggesting that SARS-CoV-2 penetration across the GI organs may impose risks to mucosal integrity. IBD, comprising Crohn’s disease (CD) and ulcerative colitis (UC), is a chronic inflammatory disease of the GI tract [32,64]. An earlier study reported a higher expression of ACE2 in the inflamed gut of patients with IBD [65]. In contrast, during early COVID-19 outbreaks in Wuhan (China), no SARS-CoV-2 infection was reported in a cohort of 318 registered IBD patients (204 UC patients and 114 CD patients) [66]. The exact reason for this observation is not entirely clear, but a proper communication with IBD patients regarding hygiene and restricted use of immunomodulators might relate to no COVID-19 symptoms. Moreover, this could also be due to less reporting of IBD cases vs. non-IBD cases, or IBD patients not showing symptomatic COVID-19 infection. IBD patients are usually prescribed anti-inflammatory compounds in the course of treatment, and it is possible that frequent use of immunomodulators to treat COVID-19 patients may interfere with IBD clinical manifestations [67]. It should be noted that ACE2 receptor expression is detected in the cells of terminal ileum and colon, and these GI locations are more susceptible to IBD-associated inflammation [68,69]. Although, limited reports of IBD pathogenesis in the context of COVID-19 are available, the outcomes of these studies indicate a possible GI and mucosal alteration in COVID-19 patients, and further studies are needed to address various pressing questions to provide better treatment and care.

## 3. Vaccine Platforms

The spike (S) protein is a key antigenic target for COVID-19 vaccine development [70]. It is noteworthy that the SARS-CoV-2 spike protein induces robust CD4+ T cell response which correlates with the magnitude of the anti-SARS-CoV-2 IgG and IgA titers [71]. Furthermore, in mice models and in human clinical trials, vaccines encoding SARS-CoV-2 S protein elicited both humoral and cellular immune responses required to neutralize infection [72].

Based on several different development platforms, multiple vaccine candidates (>280) have been identified to target COVID-19. Currently, over 100 COVID-19 vaccine candidates are under clinical development in an unprecedented expeditious development effort. Protein-based vaccines constituted the largest category, accounting for 31% of all vaccine candidates being developed [73]. Other vaccines are based on viral vectors, nucleic acids, inactivated virus, live attenuated virus and virus-like particles, which account for 21%, 26%, 16%, 2%, and 5%, respectively [73].

### 3.1. Protein-Based Vaccines (PV)

Protein-based vaccines are produced by recombinant DNA (rDNA) technology approach to express viral surface protein (either the full-length S protein or its RBD domain) in different host expression systems (such as *Escherichia coli*, yeasts, insect cells and mammalian cells) and are capable of eliciting antigenicity in the host immune system [74]. It is noteworthy that vaccines based on this technology include hepatitis B, influenza (FluBlok), human papilloma virus (HPV) and meningococcal B that are currently in the market. Several COVID-19 protein-based vaccines are in the advanced stage of clinical development (Table 1). A PV based vaccine developed by Novavax, the NVX-CoV2373 (a nanoparticle-based recombinant pre-fusion S protein) is currently under Phase 3 clinical trial, and demonstrates an efficacy rate of 96% against SARS CoV-2 [75,76]. Interestingly, compared to other vaccine types (i.e., viral vector-based and mRNA vaccines), NVX-CoV2373 reportedly generates higher titers of total as well as neutralizing antibodies against SARS CoV-2 virus [75,77,78,79]. Furthermore, combination with Matrix-M1 adjuvant induces polyfunctional CD4+ T-cell response as reflected by increased levels of IL-2, IFN- γ, TNF- α production [80]. Another peptide-based COVID-19 vaccine, UB-612 (developed by Vaxxinity), is the first ‘multitope’ (derived from RBD, the S2 protein, as well as membrane and nucleoprotein regions of the SARS-CoV-2 virus) vaccine that generates higher neutralizing antibody titers that exceed those in human convalescent serum [81]. Notably, UB-612 induces neutralizing antibodies in 100% of participants in Phase 1 clinical trial [82].

Recombinant protein-based vaccines offer distinct advantages over other vaccine platforms [83]. They induce a safe and robust immune response (along with adjuvants), are easy to generate and require much less stringent storage and distribution requirements than mRNA vaccines [84,85]. In addition, unlike the viral vector-based vaccines, they do not carry the risk of pre-existing adenoviral immunity. However, a major limitation with PV is difficulty in expressing membrane-bound spike protein, which is likely to affect production yields [86]. In addition, there are concerns over adverse immune reactions triggered by full-length spike protein [87]. Although RBD is a relatively small protein and easier to purify, it lacks other neutralizing epitopes, rendering RBD-based vaccines less effective than the full-length version [88]. In addition, protein-based vaccines are typically via intramuscular (IM) injection and are not expected to result in robust mucosal immunity [88].

Virus-like particle (VLP)-based vaccines are a subset of protein vaccines that constitute some or all of the proteins derived from the viral capsid, which can then self-assemble into the virus-like structure [89]. Since VLP cannot replicate, they provide a safer alternative to live-attenuated vaccines (LAV). All four FDA-approved vaccines for hepatitis B and HPV, are based on highly purified VLP [90]. Currently, three VLP candidates are in clinical development to target COVID-19 (Table 1).

### 3.2. Nucleic Acid Vaccines

Nucleic acid-based vaccines such as mRNA and DNA vaccines, encode the genetic instruction to synthesize protein antigen using the host cell translational machinery. Such a platform offers great flexibility in manipulating the coded antigen, which, in turn, shows great potential for rapid production. Nucleic acid vaccines can be classified further into mRNA and DNA-based vaccines.

#### 3.2.1. mRNA Vaccines

mRNA vaccines comprise an RNA molecule, encapsulated in lipid nanoparticles (LNPs). Following intramuscular injection, LNP-mRNA is internalized in the host cells and serves as a template to synthesize full-length spike protein antigen. mRNA vaccines have multiple advantages over conventional approaches in terms of safety, cost effectiveness and induction of both cell and antibody mediated immune response [91,92].

Two mRNA vaccines, from Pfizer/BioNTech (BNT162b2) and Moderna (mRNA-1273), have already been granted emergency use authorization (EUA) in multiple countries. Both BNT162b2 and mRNA-1273 demonstrate a vaccine efficacy of 95% and 94.1%, respectively, in preventing COVID-19 disease [93,94]. mRNA-1273 vaccine had 100% efficacy against severe COVID-19 illness [95]. Noteworthy, both BNT162b2 and mRNA-1273 vaccines induce a higher GMT (Geometric Mean Titer) and PRNT80 (Plaque Reduction Neutralizing Testing) value compared to convalescent serum panel. Furthermore, both the mRNA vaccines induce a robust CD4+ T cell response in almost all recipients [80]. For solid-organ transplant recipients, administration of a third dose of the BNT162b2 vaccine significantly improved the immunogenicity of the vaccine, with no cases of COVID-19 reported in any of the patients [96]. Interestingly, a clinical trial with more than 600 people who already received first dose of the ChAdOx1 nCoV-19 (developed by Oxford–AstraZeneca, an adenovirus-based vaccine) followed by a booster (eight weeks later) of BNT162b2, showed robust humoral response compared to single dose ChAdOx1 nCoV-19 vaccine. These antibodies were able to recognize and inactivate SARS-CoV-2 in laboratory tests [97]. Another mRNA vaccine, CVnCoV (developed by CureVac AG), effectively generates neutralizing antibodies against SARS-CoV-2 as observed in convalescent patient sera [98]. Notably, CVnCoV vaccine is under Phase 3 clinical trial (Table 2) and stable for at least three months when stored at 5°C [99]. Preliminary data from Phase 3 study (40,000 person trial) showed that the CureVac’s mRNA vaccine was only 47% effective at preventing COVID-19 severity [100].

#### 3.2.2. DNA Vaccines

DNA vaccines are based on plasmid DNA constructs containing mammalian expression promoters and a transgene encoding immunogenic spike protein antigen. Compared with traditional approaches, DNA vaccines have several advantages, such as induction of broad immune responses, thermal stability, possibility of encoding multiple antigens in a single vaccine, efficient large-scale production in bacteria and cost effectiveness [101].

Inovio Pharma has designed a COVID-19 vaccine candidate (INO-4800) encoding a full-length S protein with an N-terminal IgE leader to increase immunogenicity (Table 2). INO-4800 mode of administration is intradermal and induces neutralizing antibodies that block SARS-CoV-2 S protein binding to the host ACE2 receptor [102]. Furthermore, DNA vaccines ZyCov-D (Zydus Cadila, Ahmedabad India) and AG0301 (Osaka University, Osaka, Japan) are currently in late-stage clinical trials (Table 2).

### 3.3. Viral Vector-Based Vaccines

Viral vector-based vaccines consist of a genetically modified virus (i.e, the vector) to express foreign antigen(s) using the host translational machinery. Adenovirus, measles, lentivirus and vesicular stomatitis virus (VSV) vectors are commonly used vector designs for vaccine development. Viral vector vaccines can be broadly classified into a non-replicating viral vector and replicating vector vaccines.

#### 3.3.1. Non-Replicating Viral Vector Vaccines

Adenoviral-based vector is the most widely used and advanced vector candidate for the non-replicating viral vaccine design. They are typically rendered replication ineffective due to the deletion of virus structural genes (E1 and E3), thus no new virus particles are formed [103]. Non-replicating vectors have been engineered to produce the encoded antigen, i.e., spike/RBD protein, hence both humoral and cellular immune responses are stimulated. However, one of the major disadvantages is that some of these vectors are partially neutralized due to pre-existing vector immunity, thus reducing the vaccine efficacy [104].

Multiple non-replicating vector vaccine candidates have progressed in clinical development (Table 3). To avoid pre-existing immunity concerns, a modified version of a chimpanzee adenovirus known as ChAdOx1, was designed by the University of Oxford/AstraZeneca to develop and test a ChAdOx1 nCoV-19 vaccine [105]. Upon vaccination, robust B cell activation and proliferation was observed with antibody production predominantly of IgG1 and IgG3 subtype. Furthermore, this vaccine induces broad and robust T cell response as demonstrated by an increase in the level of IFN-γ, TNF- α and IL-2 secretion [106]. Overall, the vaccine offers strong protection, with an overall efficacy of 76% [107]. This vaccine is approved in Brazil and authorized for emergency use in multiple countries. JNJ-78436735, developed by Johnson & Johnson, demonstrated that a single dose of the vaccine had an efficacy rate of 72% in the United States and lower efficacy in countries where more contagious variants are widespread [108]. The vaccine has been authorized for emergency use by the European Union, the United States and other countries. A recent study suggests that vaccine-induced immune thrombotic thrombocytopenia (VITT) (a side effect linked to Oxford/AstraZeneca and Johnson & Johnson) could be overcome by using re-optimized spike protein open reading frame to avoid generating unintended splice protein variants and thereby increasing the safety of adenoviral-based COVID-19 vaccines [109].

Sputnik V, or Gam-COVID-Vac, developed by Gamaleya Research Institute (Russia), uses two different adenoviruses, Ad26 and Ad5, to overcome pre-existing adenoviral immunity. It is a two-dose vaccine with an efficacy rate of 91.6% [110]. The vaccine is approved in Russia and is authorized for emergency use in multiple countries. Recently, a single-dose version dubbed “Sputnik Light” was authorized for emergency use in Russia with an efficacy of 79.4% [111].

#### 3.3.2. Replicating Viral Vector Vaccines

Replicating viral vector vaccines can propagate themselves in host cells such that a lower dose might be able to induce robust immune response [88]. Beijing Wantai Biological Pharmacy is developing a COVID-19 vaccine based on intranasal flu-based-RBD and is in Phase 2 trial (Table 3). Multiple vaccine candidates, including vectors based on lentivirus, vesicular stomatitis virus (VSV) and Newcastle disease virus (NDV), are in clinical development [112,113]. NDV vector has several advantages as it is safe in humans and can be amplified in embryonated chicken eggs, thereby allowing for high yields, low cost per dose and offering an intranasal route of administration [88].

### 3.4. Inactivated Vaccines (IVs)

IVs are generated by chemical neutralization (typically by beta-propiolactone) of the SARS-CoV-2 virus, propagated using Vero cell lines in conditional medium [114]. IVs have been traditionally effective against polio, rabies and hepatitis A, and showed promising antibody titers against SARS-CoV-2 compared to other vaccine types [115]. Several IV candidates are under clinical trials, with CoronaVac (developed by Sinovac) currently in the advanced stage of clinical development (Table 4). The vaccine is approved for use in China and has EUA in multiple countries [116]. In Brazil, researchers found it had an efficacy of 50.65% [116]. In addition, COVAXIN (developed by Bharat Biotech, India) received authorization for emergency use in India, and trial results demonstrated an efficacy rate of 78% against mild, moderate and severe COVID-19 disease [117]. The advantage of this approach is that the inactivated virus in these vaccines cannot undergo an active replication cycle and are usually considered safer than live-attenuated vaccine constructs. Inactivated viruses are likely to elicit an immune response, not only against the S protein of SARS-CoV-2, but the entire virus since the whole inactivated virus is presented to the immune system. However, manufacturing is time-consuming and requires a biosafety level 3 facility [88].

### 3.5. Live-Attenuated Vaccines (LAV)

Live-attenuated vaccines are produced by generating a weakened version of the virus with limited replication capacity, yet still retain the ability to induce an antiviral immune response comparable to natural infection [118]. These vaccines typically induce both antibody and cell-mediated immune responses [84]. A key advantage with LAVs is that they can be administered intranasally to induce a mucosal immune response to protect the upper respiratory tract, which is the primary entry site for SARS-CoV-2 virus replication. Currently, there are two candidates in clinical development (COVI-VAC and MV-014-212), both of which use the codon de-optimization approach for virus attenuation [119,120] (Table 4).

## 4. Pharmacological Therapies

Currently, there is no effective antiviral therapy for treating SARS-CoV-2 infection. Pharmacological therapies are recommended based on the understanding of the COVID-19 disease progression. Typically, antiviral therapies (e.g., Remdesivir) would have the greatest effect early in the disease course, primarily driven by SARS-CoV-2 virus replication. Later in the disease course, when immune/inflammatory response is amplified, immunosuppressive/anti-inflammatory therapies (e.g., Dexamethasone, Baricitinib) are likely to be more beneficial to have a clinical impact.

### 4.1. Remdesivir

Remdesivir is an FDA-approved broad-spectrum antiviral drug made by Gilead Sciences for COVID-19 treatment (Table 5). It is an intravenous nucleoside analog and inhibits the RNA-dependent RNA polymerase (RdRp) of SARS-CoV-2, thus prematurely terminating viral replication [121,122]. Multiple in vitro studies demonstrated that, at the nanomolar concentration, remdesivir is effective against SARS-CoV-2 infection [123,124,125]. Interestingly, a recent in vitro study revealed that a combination of remdesivir with repurposed hepatitis C virus (HCV) drugs was 10 times more effective at inhibiting SARS-CoV-2 [126]. In addition, in non-human primate studies, remdesivir treatment had a clinical benefit as reflected by reduced viral load and lung damage [127].

Currently, multiple clinical trials are ongoing to evaluate the safety and efficacy of remdesivir in COVID-19 patients. A recent study demonstrates a 11 day median recovery time compared with 15 days for hospitalized COVID-19 patients receiving placebo, and while not statistically significant, remdesivir also helped to reduce mortality [128]. Yet, many clinicians remain skeptical of remdesivir’s benefits. A recent meta-analysis study of randomized controlled trials demonstrates no statistically significant evidence of reduced mortality in hospitalized COVID-19 patients when treated with remdesivir [129]. On 19 November 2020, WHO recommended against using remdesivir. Based on the Solidarity Trial Result in Feb 2021, it was concluded that remdesivir had little to no effect on hospitalized COVID-19 patients [130]. Currently, as per National Institute of Health (NIH) COVID-19 treatment guidelines, there is insufficient data to recommend remdesivir for routine use. [131].

### 4.2. Dexamethasone

Dexamethasone is a glucocorticoid with potent anti-inflammatory properties. It is approved for the treatment of multiple inflammatory diseases [132]. Dexamethasone inhibits the production of pro-inflammatory cytokines such as interleukin IL-1, IL-2, IL-6, IL-8, VEGF, TNF, prostaglandins and IFN-gamma. Importantly, increased amounts of such cytokines are linked to COVID-19 disease severity. At the same time, it can also induce activation of anti-inflammatory cytokine synthesis, notably IL-10 and lipocortin-1 [133]. Due to their immunosuppressive and potent anti-inflammatory effect, glucocorticoids have been widely used to treat COVID-19 related syndromes, like SARS, Middle East respiratory syndrome (MERS), severe influenza and acute respiratory distress syndrome (ARDS) [134,135,136]. The Randomized Evaluation of COVID-19 Therapy (RECOVERY) trial, a multicenter, open-label trial in hospitalized COVID-19 patients, indicated that the use of dexamethasone compared to standard of care reduced 28 days mortality (endpoint) in patients requiring oxygen therapy and/or ventilation support [137]. In addition, a prospective meta-analysis of seven randomized trials showed that 28 day all-cause mortality was lower among patients who received corticosteroids compared with those who received usual care or placebo [138]. Based on the current evidence, the NIH COVID-19 treatment guideline recommends using 6mg of dexamethasone per day, up to 10 days or until hospital discharge in COVID-19 patients requiring ventilation and/or supplemental oxygen. [139]. It should be noted that corticosteroids may be less likely to benefit COVID-19 patients with more recent symptom onset [131].

### 4.3. Favipiravir

Favipiravir is an oral antiviral compound that is effective against a broad spectrum of RNA viruses [140,141]. It has been approved to treat influenza in Japan under the brand name Avigan. Favipiravir is a selective and potent inhibitor of viral RNA polymerase [142]. A report from Wang et al. showed favipiravir inhibits (EC50: 61.88 μM) SARS-CoV-2 infection in Vero cells [124]. In addition, a recent clinical study (N = 80) with mild to moderate COVID-19 patients revealed that treatment with favipiravir led to shorter viral clearance time (4 vs. 11 days) and a significant improvement rate in chest imaging (CT) (91.43% vs. 62.22%) were observed compared to the control arm [143]. Furthermore, a meta-analysis of nine clinical studies showed significant clinical improvement in the favipiravir group versus the control group as reflected by viral clearance rate, requirement for oxygen therapy, ICU transfer and reduced mortality [144]. Favipiravir is approved for COVID-19 in China, India, Japan and Russia (Table 5).

### 4.4. Opaganib

Opaganib is a novel, orally administered sphingosine kinase-2 (SK2) selective inhibitor developed by RedHill Biopharma for treating COVID-19 patients. Opaganib demonstrated potent SARS-CoV-2 antiviral activity by completely inhibiting viral replication in vitro human lung tissue model. Phase 2 proof-of-concept study (*n* = 40) with opaganib (NCT04414618) showed a consistent trend of more remarkable improvement in reducing oxygen requirement compared to the placebo arm [145].

### 4.5. Tocilizumab

Tocilizumab is a humanized anti-IL-6 receptor monoclonal antibody that is FDA-approved for rheumatologic disorders, giant cell arteritis, Castleman’s disease and cytokine release syndrome (CRS) associated with chimeric antigen receptor T cell (CAR T-cell) cancer therapy [146,147,148]. Previous clinical trials have so far shown mixed results for 28 day mortality. Six trials reported no benefit, while the Randomized, Embedded, Multifactorial Adaptive Platform Trial for Community-Acquired Pneumonia (REMAP-CAP) trial reported improved outcomes, including survival in critically ill COVID-19 patients requiring respiratory or cardiovascular organ support [149,150]. Based on the current evidence, the FDA Panel recommends using tocilizumab (single intravenous dose of 8 mg/kg) in combination with dexamethasone (6 mg daily for up to 10 days) in hospitalized patients exhibiting rapid respiratory decompensation due to COVID-19 [151].

### 4.6. Chloroquine (CQ) and Hydroxychloroquine (HCQ)

CQ and HCQ are antimalarial drugs and have also been approved to treat autoimmune diseases, such as systemic lupus erythematosus (SLE) and rheumatoid arthritis [152]. Although the possible mechanism of action is not yet fully understood, it is believed that both drugs elevate the pH of intracellular organelles, such as endosomes/lysosomes, thereby impeding fusion and uncoating and, ultimately, viral replication [153]. Initial in vitro tests demonstrate inhibitory effects of CQ and HCQ on SARS-CoV-2 replication and infection [154,155]. However, such in vitro effects are not replicated in SARS-CoV-2 infection model studies in hamsters and non-human primates [156]. Furthermore, multiple reports from clinical trials investigating the potential therapeutic safety and efficacy of CQ and HCQ demonstrate no significant difference in the rate of viral clearance, disease progression and 28 day all-cause mortality in mild to moderate COVID-19 patients [157,158,159]. Considering the lack of a benefit seen in the clinical trials and the potential for toxicity, FDA recommends against using CQ/HCQ to treat hospitalized COVID-19 patients [160].

### 4.7. Baricitinib

Baricitinib is an oral (Janus kinase) JAK inhibitor and is FDA approved for the treatment of rheumatoid arthritis [161]. Baricitinib is a selective JAK1 and JAK2 inhibitor and inhibits JAK1/2-dependent cytokines (e.g., IL-6 and interferon [IFN]-γ), typically involved in COVID-19 inflammation [162]. The anti-inflammatory and antiviral activity of baricitinib was demonstrated by its ability to reduce viral infectivity in human primary liver spheroids and in COVID-19 patients exhibiting a rapid decline in viral load, inflammatory markers and IL-6 levels [163]. Initial reports from a multicenter, randomized, double-blind ACTT-2 trial showed that baricitinib plus remdesivir was superior to remdesivir alone in reducing recovery time by about a day, with no impact on mortality and accelerating clinical status improvement in hospitalized COVID-19 patients receiving high-flow oxygen or noninvasive ventilation [164]. Baricitinib and dexamethasone are the only two therapies that reduce inflammation and have demonstrated efficacy in clinical trials to treat hospitalized COVID-19 patients.

### 4.8. SARS-CoV-2 Monoclonal Antibodies

Most of the antiviral monoclonal antibodies (mAbs) under development target the surface spike protein of SARS-CoV-2 [165]. Currently, Regeneron Pharmaceuticals REGEN-COV (Casirivimab with Imdevimab), Eli Lilly (Bamlanivimab and Etesevimab) and Vir Biotechnology/GlaxoSmithKline (Sotrovimab), have been authorized for emergency use by FDA to treat mild to moderate non-hospitalized COVID-19 patients.

REGEN-COV (Casirivimab with Imdevimab) contains two different monoclonal antibodies that bind to unique epitopes of the spike protein RBD of SARS-CoV-2. In a randomized, double-blinded, placebo-controlled Phase 2 clinical trial (*n* = 799), significant reductions were observed in the level of the virus along with fewer medical visits within 28 days of receiving the REGEN-COV treatment compared to placebo [166].

Bamlanivimab (also known as LY-CoV555 and LY3819253) is a neutralizing monoclonal antibody that targets the RBD of the S protein of SARS-CoV-2. Etesevimab (also known as LY-CoV016 and LY3832479) is another neutralizing monoclonal antibody that binds to a different but overlapping epitope in the RBD of the SARS-CoV-2 S protein. Initial reports from the randomized phase 2/3 BLAZE-1 clinical trial (*n* = 577) showed that the treatment with a combination arm (Bamlanivimab + Etesevimab) significantly decreased SARS-CoV-2 log viral load at day 11 compared with placebo in mild to moderate COVID-19 patients [167].

Sotrovimab is a single dose monoclonal antibody targeting the spike protein of SARS-CoV-2, thereby blocking virus attachment and entry into human cells. Interim analysis from Phase 3 COMET-ICE (COVID-19 Monoclonal antibody Efficacy Trial–Intent to Care Early) trial (*n* = 583) demonstrated an 85% reduction in hospitalization or death in patients receiving sotrovimab (monotherapy) compared to placebo in mild-to-moderate COVID-19 patients [168].

### 4.9. Convalescent Plasma Therapy (CPT)

CPT is a passive immunization approach to treat infectious diseases using plasma with high antibody titer which, in principle, could stop the disease progression. CPT is considered standard of care for the treatment of Argentine hemorrhagic fever [169]. In addition, multiple nonrandomized trials have claimed efficacy in SARS, MERS, H1N1 influenza, and Ebola [170]. Given the lack of effective therapeutic strategy against SARS-CoV-2, CPT may be an essential tool for treating COVID-19 patients. Multiple randomized trials of convalescent plasma for the treatment of hospitalized patients with COVID-19 have been reported, however, none of these trials have demonstrated a beneficial effect on mortality [171,172]. Recently, FDA revised to limit the authorization of high-titer convalescent plasma only in early disease stage COVID-19 hospitalized patients [173].

### 4.10. Additional Novel Therapies

#### 4.10.1. Inhaled Nanobodies

Compared to mAbs, camelid single-domain antibody fragments called nanobodies are cost-effective and exhibit unique biophysical properties, including small size and stability, allowing efficient pulmonary administration via aerosolization [174]. A recent study demonstrated that low doses (0.2 mg/kg) of an aerosolized nanobody named Pittsburgh inhalable Nanobody-21 (PiN-21), protected against moderate to severe COVID-19 infection in Syrian hamsters model, from infection-induced weight loss, decreased lung viral titers by a million-fold and prevented lung damage compared to placebo treatment that did not neutralize the virus [175]. It remains to be seen whether such therapeutic benefits can be translated in human clinical trials.

#### 4.10.2. Mesenchymal Stem Cells Therapy

Mesenchymal stem cells (MSCs) are multipotent adult stem cells and have immunomodulatory and immune-privileged potential. Furthermore, MSCs lack ACE2 receptors and TMPRSS2, making them resistant to SARS-CoV-2 infection [176]. In a recent pilot study of seven patients with COVID-19 pneumonia, intravenous administration of clinical-grade human MSC showed improved functional outcomes and recovery compared to the placebo arm [177]. At present, multiple clinical trials (+70) are evaluating the efficacy of MSCs for COVID-19 treatment.

**Table 5:** Promising COVID-19 therapeutic drugs in clinical development.

Manufacturer	Name	Target	Mechanism of Action	Phase	RoA
Gilead Sciences Inc	Veklury (Remdesivir)	Viral RNA polymerase	Inhibitor of viral replication	Approved	IV
None (generic)	Dexamethasone	Glucocorticoid receptor agonist	Alters the body’s normal immune system responses	Approved	Oral
Fujifilm Toyama Chemical	Favipiravir	Viral RNA polymerase	Inhibitor of viral replication	Approved (as generics)	Oral
Eli Lilly	Olumiant (Baricitinib)	JAK1/2 inhibitor	Decreases immune system activation	EUA	Oral
Regeneron/Sanofi	Casirivimab and Imdevimab (REGN-COV2)	Viral epitopes	Binds to virus and neutralizes its ability for infection	EUA	IV
Eli Lilly	Bamlanivimab (LY-CoV555) and Etesevimab (LY-CoV016)	Viral epitopes	Binds to virus and neutralizes its ability for infection	EUA	IV
GSK/ Vir Biotech	Sotrovimab	Viral epitopes	Binds to virus and neutralizes its ability for infection	EUA	IV
Roche/Chugai	Tocilizumab (Actemra)	IL-6	Decreases immune system activation	Phase 3	IV and SC
Sanofi	Chloroquine/Hydroxychloroquine	Endosomal vesicles	Antiviral activity through pH change	Phase 3	Oral
Humanigen [178]	Lenzilumab	GM-CSF	Neutralizes circulating GM-CSF	Phase 3	IV
RedHill [145]	Opaganib	Sphingosine kinase-2 (SK2)	SK2 inhibitor	Phase 3	Oral
EUSA Pharma [179]	Siltuximab	IL-6	Decreases immune system activation	Phase 3	IV
Merck [180]	MK-4482	Viral RNA polymerase	Inhibitor of viral replication	Phase 3	Oral
Synairgen [181]	SNG001	IFN-beta-1a	Delivery of FN-beta inhibits viral replication	Phase 3	IN
GSK/Vir [168]	GSK4182136	Viral epitopes	Binds to virus and neutralizes its ability for infection	Phase 3	IV, IM
PharmaMar [182]	Plitidepsin (Aplidin)	eEF1A	eEF1A inhibitor	Phase 2	IV
Pfizer [183]	PF-07321332	3CL protease	3CL protease inhibitor	Phase 1	Oral

RoA, route of administration; IV, intravenous; IN, intranasal; SC, subcutaneous.

## 5. SARS-CoV-2 Variants

Genomes of coronaviruses such as SARS-CoV-2, can alter their genome sequence during replication in host cells, referred to as mutations. A population of coronaviruses that inherit the same distinctive mutations is called a variant. Several mutations and variants of SARS-CoV-2 have arisen throughout the world over the course of the pandemic. From the evolutionary perspective, those variants that confer a competitive advantage with respect to viral replication, viral transmission or escape from immunity are most likely to increase in frequency. However, chance events, chronic infection in immunosuppressed individuals and host shifts could also increase the frequency of a particular strain. RNA viruses such as SARS-CoV-2 mutate more slowly than most RNA viruses due to proofreading function during replication, which results in fewer mutations and higher accuracy in virus replication [184]. SARS-CoV-2 variants first started emerging in early March 2020 having a single D614G mutation in the spike (S) glycoprotein, and variants having this mutation predominated since June of 2020 [185], possibly due to enhanced viral fitness and transmissibility [186,187]. Although several vaccine constructs have shown promising outcomes, including BNT162b2 and mRNA-1273 with more than 95% protective efficacy against COVID-19 [93,188], these interventions were directed towards the initial SARS-CoV-2 virus that emerged in 2019. The recent emergence of new SARS-CoV-2 variants is a matter of concern due to several mutations that have arisen in the spike protein. Such mutations could impact the structure of the protein, thereby altering infection rates by modifying the interaction of the spike protein with the human hACE2 receptor, modifying immune response, or compromising the efficacy of treatments by monoclonal antibodies. The World Health Organization has classified variants as ‘Variants of Interest’ (VOI) and ‘Variants of Concern’ (VOC). We will discuss the important SARS-CoV-2 VOCs in this section.

### 5.1. The B.1.1.7 Lineage (Alpha Variant)

The B.1.1.7 variant is also known as 20I/501Y.V1. B.1.1.7 was first detected in the United Kingdom in December 2020 and was named VOC 202012/01 since it quickly surged in other countries at an exponential rate [189]. Coronaviruses from the B.1.1.7 lineage are between 40% to 83% more infectious than the wild type B1 strain, result in higher nasopharyngeal viral loads and cause more serious disease [190,191,192]. The B.1.1.7 lineage has now been detected in over 50 countries, including the United States.

The high infection rate of B.1.1.7 is attributed to several mutations in its spike protein, including two deletions, namely H69/V70, and Y144/145, and six substitutions, including N501Y, A570D, P681H/R, T716I, S982A and D1118H. The key mutations of B.1.1.7 that highly affect transmissibility, disease severity and infection rate are N501Y, H69/V70 deletion and P681H/R (Figure 2B). The H69/V70 deletion results in a two-fold increase in S protein-mediated infectivity in vitro using pseudotyped lentivirus [193]. This deletion is speculated to modify the immunodominant epitopes located at variable loops within NTD, conferring resistance to neutralization by sera from both convalescent patients and vaccinated individuals [194]. The Y144/145 deletion occurs on the edge of the spike tip and is speculated to modify binding of antibodies to SARS-CoV-2. The N501Y mutation is in the RBD of the spike protein and is thought to be critical in increasing virus transmission since it helps the virus to increase binding to hACE2 receptors, which are found on the membranes of the human heart, kidney and lung cells [195,196]. The N501Y mutation has also been linked to increased infectivity and virulence in mouse and ferret models [63]. Another key mutation is in the P681 residue, which is adjacent to the furin cleavage site that separates S1 and S2 subunits of the S protein. The P681H and P618R mutations facilitate easier access of the human proteases to the furin cleavage site, thus increasing SARS-CoV-2 transmission and infection [197,198].

Recent studies have demonstrated reduced but, overall, largely preserved neutralizing titers using pseudoviruses with the complete set of mutations described for B.1.1.7 variant [199,200]. Correspondingly, modest reductions in the neutralizing activity of both plasma from convalescent patients (2.7–3.8-fold) and sera from individuals that received Moderna or Pfizer vaccines (1.8–2-fold) have been observed [201,202]. The Gam-COVID-Vac Sputnik V vaccine sera effectively neutralized B.1.1.7. viruses, albeit with highly variable titers, while NVX-CoV2373 demonstrated an efficacy rate of 86.3% against mild, moderate and severe COVID-19 caused by B.1.1.7 as compared to 96% efficacy seen in the wild type B1 strain [76,203]. Overall, although there are variations in study design, the efficacy of currently available vaccines is either similar or moderately lower against the B.1.1.7 variant (Table 6). The B.1.1.7 variant also retains in vitro susceptibility to the anti-SARS-CoV-2 monoclonal antibodies that are currently available through Emergency Use Authorization (EUA) [204] (Table 6).

### 5.2. The B.1.351 Lineage (Beta Variant)

This variant is also known as 20H/501Y.V2 and was first identified in South Africa in December 2020, with samples dating back to the beginning of October 2020 [205]. This variant has enhanced transmissibility and is designated as a VOC since it has been detected outside of South Africa, including in the United States.

B.1.351 shares the N501Y mutation with B.1.1.7 in the RBD domain of the spike protein (Figure 2B). This variant also has two additional mutations in the same RBD domain (K417N and E484K) that play a pivotal role in both the interaction with the receptor and immune evasion. Compared to the Wuhan reference strain, the B.1.351 variant has 12 non-synonymous mutations and one deletion. B.1.351 contains nine mutations in the spike protein including L18F, D80A, D215G, LAL 242–244 del, R246I, K417N, E484K, N501Y, D614G and A701V, while the remaining ones are located in ORF1a [K1655N], envelope (E) [P71L] and N [T205I] viral proteins. Out of the nine spike mutations, LAL 242-244 del & R246I mutations are in the NTD, while K417N, E484K & N501Y are in RBD, and A701V is located near the furin cleavage site [205]. Nelson et al., using molecular dynamic simulation, have demonstrated that the E484K mutation enhances spike RBD-ACE2 affinity and the combination of E484K, K417N and N501Y mutations in the B.1.351 variant induce conformational changes greater than the N501Y mutant alone, resulting in an escape mutant [206].

VSV pseudoviruses with spike containing K417N-E484K-N501Y-D614G and full B.1.351 mutations resulted in 2.7- and 6.4-fold Geometric Mean Titer (GMT) reduction, respectively, as compared to the D614G VSV original isolate. In addition, sera from the Moderna and Pfizer-BioNTech vaccines show significantly reduced neutralization of B.1.351 (12.4 fold, Moderna; 10.3 fold, Pfizer) [202]. The Gam-COVID-Vac Sputnik V vaccine sera exhibited moderate and markedly reduced neutralization titers against E484K and B.1.351 variants, respectively [203]. Serum samples obtained after the second dose of the BBIBP-CorV vaccine (Sinopharm), or CoronaVac vaccine serum samples, showed complete or partial loss of neutralization against B.1.351 [207]. Finally, randomized placebo-controlled clinical trials reported in a press release by Novavax and Janssen companies in South Africa indicate significant decrease in the efficacy of their vaccines in places where the B.1.351 variant dominated [97,208]. Similarly, a clinical trial evaluating two dose regimen of AZD1222 (AstraZeneca/Oxford vaccine) in South Africa did not show protection against mild to moderate COVID-19 due to B.1.351 variant [97,209]. In Qatar, mass immunization campaigns have revealed that the estimated effectiveness of the Pfizer-BioNTech vaccine against the B.1.1.7 variant was 89.5% at 14 or more days after the second dose, while the effectiveness against infection with the B.1.351 variant was 75.0% [210]. Overall, the BNT162b2 (Pfizer-BioNTech) vaccine was effective against infection and disease in the population of Qatar, despite the B.1.1.7 and B.1.351 variants being predominant within the country. However, vaccine effectiveness against the B.1.351 variant was approximately 70%, which is lower than the effectiveness (>90%) reported in the clinical trial [93] and in real-world conditions in Israel [211] and the United States [212].

B.1.351 is resistant to a major group of potent monoclonal antibodies that target the RBM, including three authorized for emergency use [201,213] (Table 3). In vitro studies suggest that bamlanivimab plus etesevimab has markedly reduced activity against the B.1.351 variant. Casirivimab activity is also significantly reduced in this variant, possibly due to the K417N and E484K mutation, although the combination of casirivimab and imdevimab appears to retain activity [204]. The US FDA has recently revoked the EUA for bamlanivimab, because of an increasing number of reports of SARS-CoV-2 variants (having the E484K mutation) that are resistant to bamlanivimab alone, in addition to B.1.351.

### 5.3. P.1 Variant (Gamma Variant)

The P.1 variant also known as 20J/501Y.V3, is a branch of the B.1.1.28 lineage that was first detected in Brazil [214] and has become a dominant variant in Brazil [215]. The P.1 variant has accumulated 12 mutations in the spike protein, including the N501Y mutation, which is also present in B.1.1.7 and B.1.351, while L18F, K417T, E484K and D614G mutations are shared with the B.1.351 variant (Figure 2B). Overall, P.1 contains 12 spike mutations in addition to D614G, including K417T, E484K and N501Y in the RBD, L18F, T20N, P26S, D138Y and R190S in the NTD, and H655Y near the furin cleavage site [214,215,216].

Neutralizing activity for the P.1 variant among vaccinated persons was lower by a factor of 6.7 for the BNT162b2 vaccine and by a factor of 4.5 for the mRNA-1273 vaccine [217]. A study using the CoronaVac vaccine showed that the immune plasma of COVID-19 convalescent blood donors had 6-fold less neutralizing capacity against the P.1 variant than against the B-1 strain. Moreover, five months after booster immunization with CoronaVac, plasma from vaccinated individuals failed to efficiently neutralize P.1 lineage isolates [218]. However, real world data demonstrates 49.6% effectiveness of the vaccine, which is similar to the vaccine’s efficacy of 50.34% against symptomatic COVID-19 after both doses [219].

Since the P.1 variant shares three mutations in the spike RBD residues, namely K417T, E484K and N501Y with B.1.351, it is resistant to neutralization by several RBD-directed monoclonal antibodies, including three with EUA including bamlanivimab, due to presence of the E484K mutation [200,201,213]. Bamlanivimab plus etesevimab also has markedly reduced activity against the P.1 variant. In vitro studies also suggest that the K417T and E484 mutation, which is present in the P.1 variant, reduces casirivimab activity, although the combination of casirivimab and imdevimab appears to retain activity [204].

### 5.4. The B.1.617.2 (Delta Variant)

The B.1.617 lineage, also known as G/452.V3, was first identified in Maharashtra, India on the 5th October 2020 and is also referred to as a “double mutation” variant. Detailed analysis of the genome and proteins of B.1.617 reveal it arose independently in India. On the 10th of May, 2021, the World Health Organization (WHO) designated B.1.617 and its sublineages, namely B.1.617.1 (Kappa), B.1.617.2 (Delta) and B.1.617.3, as ‘Variant of Concern’. B.1.617 harbors multiple mutations in the spike protein including D111D, G142D, L452R, E484Q, D614G, P614R and P681R [211] (Figure 2B). “Double mutation” refers to B.1.617’s mutations in the SARS-CoV-2 spike protein’s coding sequence at E484Q and L452R, which are linked to increased transmission and infectivity [196,220]. The E484Q and L452R mutations confer the variant with stronger binding potential to the hACE2 receptor, as well as better ability to evade hosts’ immune systems in comparison to other variants [196,221].

The B.1.617.2 variant originally discovered in India is also known as ‘Delta’ according to the new WHO nomenclature and has already spread to at least 92 countries as of 22 June 2021. Studies suggest that Delta is 40 to 60% more contagious than the Alpha (U.K./B.1.1.7) variant and may be the most transmissible variant the world has seen as of June 2021 [222]. The Delta variant has led to a massive second wave of cases in India and has replaced the Alpha variant in the U.K. in recent months. All three sublineages harbor the L452R and the P618R mutation. The increased transmissibility of the Delta variant is attributed to the P681R mutation in the furin cleavage site, which enhances viral entry into lung cells, however Delta lacks mutations at amino acid positions 501 or 484 in its ACE2 receptor-binding domain, commonly associated with VOCs or escape from neutralizing antibodies (NAbs) [223,224]. A new version of Delta known as ‘Delta plus’ was first detected by Public Health England (PHE) on June 11th 2021. It has an additional K417N mutation which may contribute to immune escape. As of June 25th 2021, there are 200 cases associated with the Delta plus variant so far [225]. Further analysis is required to know more about its transmissibility and effect on vaccine efficacy.

According to a recent report by PHE, an analysis of 38,805 sequenced cases in England revealed that the Delta variant was associated with a 2.61 times higher risk of hospitalization within 14 days of specimen date than the Alpha variant. The Delta variant is likely to rapidly spread among unvaccinated individuals since 73% of Delta cases are seen in unvaccinated people and only 3.7% Delta cases are in people who have had both doses [226]. A recent preprint revealed that the effectiveness of BNT162b2 after two doses of vaccine reduced from 93.4% with the Alpha variant, to 87.9% with the Delta variant, while efficacy of Oxford-AstraZeneca vaccine ChAdOx1 reduced from 66.1% with Alpha to 59.8% with B.1.617.2 [217]. However, both vaccines were only 33% effective against symptomatic disease from Delta three weeks after the first dose [227]. PHE also found that Pfizer-BioNTech and the Oxford-AstraZeneca vaccine were 96% and 92% effective, respectively, at preventing hospitalization from the Delta variant [228].

Wall et al., used a high-throughput live-virus SARS-CoV-2 neutralization assay to determine the neutralization antibody titers NAbTs in 250 participants after either one or two doses of the BNT162b2 vaccine. They found that NAbTs were 5.8-fold reduced against B.1.617.2 relative to wild type on a similar order to the reduction observed against B.1.351 [224]. B.1.617 partially evaded neutralization by the antibodies induced through natural infection or immunization with the BNT162b2 and mRNA-1273 vaccine, while sera from individuals having received one dose of AstraZeneca/Oxford (ChAdOx1) vaccine barely inhibited B.1.617.2 [214,215,216]. Convalescent sera from infected patients and from recipients of BBV152 (Covaxin) were able to neutralize B.1.617 partially, but the effect was robust, as seen with mRNA vaccines [214]. Recent studies have reported resistance of B.1.617.2 to neutralization by few anti-NTD and anti-RBD mAbs, including bamlanivimab and casirivimab, attributed to the L452R, E484Q and E484K mutations [216,218,229]. Thus, B.1.617.2 spread is associated with an escape to antibodies targeting epitopes on the S protein.

### 5.5. The CAL.20C Variant

This variant discovered in California constitutes the B.1.427 and B.1.429 lineages and carries the L452R mutation, as seen in the lineage B.1.617. It is designated as a ‘Variant of Concern’ and has spread in the US and other countries [221]. It is characterized by the S13I, W152C mutations in the NTD and by the L452R mutation in the RBD (Figure 2B). The two lineages, B.1.427 and B.1.429, share the same spike protein mutations (S13I, W152C and L452R), but harbor different mutations in other SARS-CoV-2 genes. Molecular clock analysis suggest that the progenitor of both lineages emerged in May 2020, diverging to give rise to the B.1.427 and B.1.429 independent lineages in June–July 2020 [143].

Recent studies demonstrate that the average neutralization potency of the Moderna mRNA1273-elicited plasma was reduced 2.8-fold for B.1.427/B.1.429, compared to wild type (D614G) Wuhan lineage, whereas it was reduced 4-fold with Pfizer/BioNtech BNT162b2-elicited plasma [230]. The RBD L452R mutation reduces or abolishes neutralizing activity of 14 out of 35 RBD-specific monoclonal antibodies, including 3 clinical-stage mAbs, namely regdanvimab (CT-P59), etesevimab and bamlanivimab (LY-CoV555). Moreover, in vitro studies suggest a modest decrease in susceptibility to the combination of bamlanivimab and etesevimab. Due to a large structural rearrangement of the NTD antigenic supersite, there is a complete loss of B.1.427/B.1.429 neutralization for a panel of monoclonal antibodies targeting the N-terminal domain. These data suggest decreased potency of neutralization of the B.1.427/B.1.429 variant observed with vaccine elicited and infection elicited plasma results from evasion of both RBD- and NTD-specific monoclonal antibody-mediated neutralization.

### 5.6. Other Variants of Interest (VOI)

Other variants of interest include the B.1526 variant which originated in New York [231], and has the E484K mutation, and the A.23.1 mutant which has been detected in Uganda [232]. The spike mutations in B.1.526 are L5F, T95I, D253G and E484K or S477N, D614G and A701V, while those in A.23.1 include R102I, F157L, V367F, Q613H and P681R, respectively.

**Table 6:** Effect of SARS-CoV-2 variants on vaccine efficacy and therapeutics.

Name	Country of Origin	Mutations in Spike Protein	Effect on Monoclonal Antibody Treatment Regimens and Neutralization of Convalescent Sera	Effect on Vaccine Efficacy
B.1.1.7 (Alpha) ^†^	United Kingdom	N501Y *, A570D, D614G, P681H *, T716I, S982A, Δ69/70 *, Δ144 *	Retains susceptibility to EUA monoclonal antibody treatments [204] Modest reductions in the neutralizing activity of plasma from convalescent patients (2.7–3.8-fold) [201,202]	Vaccine efficacy slightly lower or unchanged, largely preserved neutralizing titers BNT162b2: 89.5–93.4% [210,233] NVX-CoV2373: 86% [234]. ChAdOx1 nCoV-19 vaccine: 70% [235,236]
B.1.351 (Beta) ^†^	South Africa	D80A, D215G, Δ241/242/243, K417N *, E484K *, N501Y *, D614G, A701V	Activity of LY-CoV555 (Bamlanivimab), and REGN10933 (Casirivimab) completely abolished [204] Significant decrease in susceptibility to the combination of bamlanivimab and etesevimab monoclonal antibody treatment [204] The combination of casirivimab and imdevimab appears to retain activity [204] Markedly more resistant to neutralization by convalescent plasma (9.4-fold) [201]	Reduced efficacy for some vaccines, completely abolished for others. BNT162b2: ~20% lower [93,211,212] Novavax vaccine: 60% [235] ChAdOx1 nCoV-19: 10% [209] Ad26.COV2.S: 52% efficacy against moderate disease, 72% efficacy against severe disease [236] Gam-COVID-Vac: abolished [203] Complete or partial loss of neutralization against BBIBP-CorV (Sinopharm) or CoronaVac (Sinovac) vaccines [207]
P.1 (Gamma) ^†^	Japan/Brazil	L18F, T20N, P26S, D138Y, R190S, K417T *, E484K *, N501Y *, D614G, H655Y, T1027I	Marked reduction in susceptibility to bamlanivimab and bamlanivimab plus etesevimab in vitro [204] Reduction in casirivimab activity, although the combination of casirivimab and imdevimab appears to retain activity [204] Reduced neutralization by convalescent and post-vaccination sera [217]. Neutralizing activity was lower by factor of: BNT162b2: 6.7 mRNA-1273: 4.5	CoronaVac: 49.6% [207] Ad26.COV2.S: Efficacy 68.1% (against moderate to severe/critical disease), 87.6% (against severe/critical disease), where P1 was detected in 30.6% of sequences [237]
B.1.617.2 (Delta) ^†^	India	L452R *, E484Q *, D614GD111D, G142D, P614R, P681R *	Abolished neutralizing activity of bamlanivimab [234] Partially evaded neutralization by the antibodies induced through natural infection [234]	BNT162b2 vaccine: 90% [233] ChAdOx1 nCoV-19: 60% [233] BBV152 (Covaxin) vaccinated individuals offer reduced but significant protection against B.1.617 as compared to B1 strain [238]
CAL.20C	California, USA	S13I *, W152C *, L452R *, D614G	Abolished neutralizing activity of Etesevimab and Bamlanivimab [204] Modest decrease in susceptibility to the combination of bamlanivimab and etesevimab [204] Reduced neutralization by convalescent and post-vaccination sera. Neutralization potency (as compared to wildtype (D614G), mRNA1273-elicited plasma reduced 2.8-fold BNT162b2-elicited plasma reduced 4 fold [230].	No evidence yet

^†^ WHO label. * Key mutations responsible for driving transmissibility and evading treatments to vaccines and therapeutics. Note: Vaccine efficacies reflect those against symptomatic infection unless otherwise specified. Vaccine efficacies between different vaccines are not to be compared directly due to variations in study design.

## 6. Conclusions

The COVID-19 pandemic has led to the development of vaccines and therapeutic regimens at an unprecedented pace. Although this pandemic has seen the emergence of several SARS-CoV-2 variants, most, if not all, vaccines have proven to be effective against them, albeit with reduced efficacy. We will continue to need more clinical data to project the long-term effects of vaccine immunity and durability on emerging variants. In conclusion, the future seems to be promising because of the extraordinary strides made in COVID-19 vaccine design and development. The next challenge before us is to make vaccines available to people from all strata of society, including those from less developed countries, so that we can finally contain the spread of SARS-CoV-2 infections.

## Figures and Tables

**Table 1:** Promising COVID-19 protein vaccine (PV) and virus-ike particle (VLP) candidates in clinical development.

Type	Manufacturer	Name	Phase	RoA	Trial Registration
PV	Novavax	NVX-CoV2373	Phase 3	IM	NCT04611802
PV	Anhui Zhifei Longcom Biopharmaceutical	SARS-CoV-2 vaccine	Phase 3	IM	NCT04466085
PV	Center for Genetic Engineering and Biotechnology (CIGB)	CIGB-66	Phase 3	IM	RPCEC00000359
PV	Federal Budgetary Research Institution State Research Center of Virology and Biotechnology “Vector”	EpiVacCorona	EUA (Russia)	IM	NCT04780035
PV	Instituto Finlay de Vacunas	FINLAY-FR-2	Phase 3	IM	RPCEC00000354
PV	Sanofi Pasteur + GSK	VAT00002	Phase 3	IM	PACTR20201152310190
VLP	VBI Vaccines Inc.	VBI-2902a	Phase 1/2	IM	NCT04773665
VLP	The Scientific and Technological Research Council of Turkey	SARS-CoV-2 VLP Vaccine	Phase 1	SC	NCT04818281
VLP	Radboud University	ABNCoV2	Phase 1	IM	NCT04839146

RoA, route of administration; IM, Intramuscular; SC, subcutaneous.

**Table 2:** Promising COVID-19 Nucleic Acid-based Vaccine candidates in clinical development.

Type	Manufacturer	Name	Phase	RoA	Trial Registration
RNA	Pfizer-BioNTech + Fosun Pharma	BNT162b2 (Comirnaty)	Approved (US, EU, Canada, Israel)	IM	NCT04760132
RNA	Moderna	mRNA -1273	Approved in Switzerland. EUA (US, EU, UK, Canada, Israel)	IM	NCT04760132
RNA	CureVac AG	CVnCoV Vaccine	Phase 3	IM	NCT04674189
RNA	Walvax Biotechnology	ARCoV	Phase 3	IM	NCT04847102
DNA	Zydus Cadila	nCov vaccine	Phase 3	ID	CTRI/2020/07/026352
DNA	Inovio Pharmaceuticals	INO-4800	Phase 2/3	ID	NCT04642638
DNA	AnGes + Takara Bio + Osaka Univ	AG0301	Phase 2/3	IM	NCT04655625

RoA, route of administration; IM, Intramuscular; ID, Intradermal.

**Table 3:** Promising COVID-19 viral vector-based vaccine candidates in clinical development.

Type	Manufacturer	Name	Phase	RoA	Trial Registration
VVnr^a^	AstraZeneca + University of Oxford	ChAdOx1-S (Covishield)	Approved (UK, India, Argentina, México)	IM	NCT04760132
VVnr^a^	CanSino Biological	Recombinant coronavirus vaccine (Ad5 vector)	EUA (Mexico)	IM	NCT04526990
VVnr^a^	Gamaleya Research Institute	Gam-COVID-Vac	EUA (Russia, Argentina, Bolivia, UAE)	IM	NCT04530396
VVnr^a^	Janssen Pharmaceutical	Ad26.COV2. S	EUA (US, Canada)	IM	NCT04505722
VVr^b^	Beijing Wantai Biological Pharmacy	DelNS1-2019-nCoV-RBD-OPT1	Phase 2	IN	ChiCTR2000039715
VVr^b^	Israel Institute for Biological Research	rVSV-SARS-CoV-2-S Vaccine	Phase 1/2	IM	NCT04608305

VVnr^a^: Viral vector non-replicating; VVr^b^: Viral vector replicating RoA, route of administration; IM, Intramuscular; IN, Intranasal.

**Table 4:** Promising COVID-19 inactivated vaccines (IV) and live-attenuated vaccine (LAV) candidates in clinical development.

Type	Manufacturer	Name	Phase	RoA	Trial Registration
IV	Sinovac	CoronaVac	Approved (China, Indonesia)	IM	NCT04756830
IV	Sinopharm	SARS-CoV-2 vaccine	Phase 3	IM	ChiCTR2000034780
IV	Sinopharm	BBIBP-CorV	Approved (China, Bahrain, UAE)	IM	NCT04863638
IV	Institute of Medical Biology + Chinese Academy of Medical Sciences	SARS-CoV-2 vaccine	Phase 3	IM	NCT04659239
IV	Research Institute for Biological Safety Problem (Kazakhstan)	QazCovid-in^®^	Phase 3	IM	NCT04691908
IV	Bharat Biotech	COVAXIN^®^	EUA (India)	IM	NCT04641481; CTRI/2020/11/028976
IV	Beijing Minhai Biotechnology	Inactivated SARS-CoV-2 vaccine	Phase 3	IM	NCT04852705
IV	Valneva, National Institute for Health Research, United Kingdom	VLA2001	Phase 3	IM	NCT04864561
LAV	Codagenix/Serum Institute of India	COVI-VAC	Phase 1	IN	NCT04619628
LAV	Meissa Vaccines	MV-014-212	Phase 1	IN	NCT04798001

RoA, route of administration; IM, Intramuscular; IN, Intranasal.

## Data Availability

The data presented in this study are available in article.
